# Prognostic factors determining poor postsurgical outcomes of mesial temporal lobe epilepsy

**DOI:** 10.1371/journal.pone.0206095

**Published:** 2018-10-19

**Authors:** Jong Hwa Shin, Eun Yeon Joo, Dae-Won Seo, Young-Min Shon, Seung Bong Hong, Seung-Chyul Hong

**Affiliations:** 1 Department of Neurology, Neuroscience Center, Samsung Biomedical Research Institute, Samsung Medical Center, Sungkyunkwan University School of Medicine, Seoul, Korea; 2 Department of Neurosurgery, Samsung Medical Center, Sungkyunkwan University School of Medicine, Seoul, Korea; University of Modena and Reggio Emilia, ITALY

## Abstract

**Objectives:**

To investigate the long-term postoperative outcomes and predictive factors associated with poor surgical outcomes in mesial temporal lobe epilepsy (MTLE).

**Materials and methods:**

We enrolled patients with MTLE who underwent resective surgery at single university-affiliated hospital. Surgical outcomes were determined using a modified Engel classification at the 2^nd^ and 5^th^ years after surgery and the last time of follow-up.

**Results:**

The mean duration of follow-up after surgery was 7.6 ± 3.7 years (range, 5.0–21.0 years). 334 of 400 patients (83.5%) were seizure-free at the 5^th^ postoperative year. Significant predictive factors of a poor outcome at the 5^th^ year were a history of generalized tonic clonic (GTC) seizures (odds ratio, OR; 2.318), bi-temporal interictal epileptiform discharge (IED) (OR; 3.107), bilateral hippocampal sclerosis (HS) (OR; 5.471), unilateral HS and combined extra-hippocampal lesion (OR; 5.029), and bi-temporal hypometabolism (BTH) (OR; 4.438). Bi-temporal IED (hazard ratio, HR; 2.186), BTH (HR; 2.043), bilateral HS (HR; 2.541) and unilateral HS and combined extra-hippocampal lesion (HR; 2.75) were independently associated with seizure recurrence. We performed a subgroup analysis of 208 patients with unilateral HS, and their independent predictors of a poor outcome at the 5^th^ year were BTH (OR; 5.838) and tailored hippocampal resection (OR; 11.053).

**Conclusion:**

This study demonstrates that 16.5% of MTLE patients had poor long-term outcomes after surgery. Bilateral involvement in electrophysiological and imaging studies predicts poor surgical outcomes in MTLE patients.

## Introduction

The rate of complete remission of seizures after epilepsy surgery for mesial temporal lobe epilepsy (MTLE) is only 60% to 70%.[1] Almost one third of patients continue to have seizures after surgery. The long-term outcome is worse than the short-term outcome, with 48 to 58 percent continuing to experience seizures 5 years after surgery.[2,3] It is important to develop reliable predictors of surgical outcomes for MTLE and selecting proper surgical candidates remains a challenge.

Clinical predictors for long-term surgical outcomes differ from the variables that predict short-term outcomes.[2–7] A history of secondarily generalized tonic-clonic seizures (SGTCS) is associated with recurrent seizures in both the short- and long-term after surgery.[4,5] The presence of SGTCS was independently associated with poor 2-year outcomes but not with 5-year outcomes;[2] in that study, epilepsy duration was the only important negative predictor for 5-year outcomes. However, the significant relationship between epilepsy duration and surgical outcome existed only in TLE with a temporal tumor or gliosis,[6] not in non-lesional TLE patients.[7]

Emerging evidence suggests that neuroimaging studies might be able to predict surgical outcomes for TLE. Gray matter abnormalities on magnetic resonance imaging (MRI) other than hippocampal sclerosis (HS), including in the entorhinal cortex and posterior parahippocampal gyrus, were a poor prognostic factor for seizure recurrence.[8] However, no statistically significant difference in surgical outcomes was found between individuals with HS and those with HS plus gray-white matter abnormalities.[9] Brain ^18F-^fluorodeoxyglucose positron emission tomography (FDG-PET) might help lateralize the seizure focus correctly and improve surgical outcomes in non-lesional TLE cases.[10] Hypometabolism that is not confined to unilateral temporal areas was associated with poor surgical outcomes in TLE patients.[11,12] Still, it is unclear whether hypometabolism extending beyond a unilateral temporal area is an independent factor predicting postoperative seizure outcomes. Patients with bilateral temporal hypometabolism (BTH) had features distinct from those with unilateral temporal hypometabolism (UTH), but their surgical outcomes did not differ.[13] Atypical hyperperfusion on single photon emission computed tomography (SPECT) was associated with poor outcomes and indicated diffuse or extra-temporal epileptogenicity.[14] Subtraction-ictal SPECT co-registered with MRI was known to enhance the diagnostic yield of epileptic foci.[15]

To the best of our knowledge, few longitudinal studies[16,17] have analyzed brain structural and functional abnormalities to find overall predictors of surgical outcomes in a large population with MTLE. We first aimed to assess poor postoperative prognostic factors in patients with MTLE using longitudinal analyses. It was hypothesized that the long-term and short-term outcomes might be affected by different sets of clinical factors. Second, we investigated whether preoperative neuroimaging results, along or in combination, predict seizure relapse after surgery.

## Methods

### Patient selection

We retrospectively reviewed the records of 868 patients with refractory epilepsy who underwent resective surgery between January 1995 and December 2011 at one university-affiliated hospital. A total of 400 patients with MTLE who underwent a standard anterior temporal lobectomy were enrolled. Epilepsy was intractable before surgery despite proper and sufficient antiepileptic drug (AED) treatment. Patients were diagnosed as having MTLE if (a) HS was seen on an MRI and mesial temporal ictal onset was identified during video-EEG monitoring, or (b) the MRI was normal or another definite pathologic lesion was found in the temporal or extratemporal regions on MRI, but video-EEG monitoring and other functional neuroimaging, including PET and SPECT, confirmed exclusive mesial temporal ictal onset. Patients with follow-up of less than 5 years were excluded. Clinical characteristics registered for each patient included age of seizure onset, age at surgery, duration of epilepsy, history of febrile seizures, SGTCS, central nervous system infections, and the existence of auras.

### Ethics statement

All patients provided written informed consent for their participation in the study. Written informed consent was obtained from the next of kin, caretakers, or guardians on the behalf of the minors/children participants involved in this study. The study was approved by Institutional Review Board of Samsung Medical Center.

### Presurgical evaluation

Intractable epilepsy patients received a comprehensive presurgical evaluation consisting of a complete neurologic examination, ictal and inter-ictal EEG results, and brain MRI during the first admission period. Ictal and inter-ictal SPECT studies[15,18,19] were performed to lateralize or localize the epileptic foci. Each patient underwent PET, a neuropsychological test, and the Wada test[13] during the second admission. All data from these admissions were reviewed and discussed in an epilepsy management conference at which surgical strategy was also discussed.

### Analysis of clinical seizures during scalp video EEG monitoring

We reviewed each patient’s seizures carefully. The presence of an aura was determined by patient’s memory or the patient pressing a button before seizures.

### Scalp video EEG monitoring

The 10/10 system for scalp electrodes was used. AEDs were usually reduced or stopped to facilitate the recording of seizures.

#### Interictal EEG classification[13]

Interictal epileptiform discharges (IEDs) were counted and analyzed over entire recording days and classified into temporal and extratemporal IED. Unilateral temporal IED was defined as strictly unilateral IED or as a 75% or more preponderance of IED in one temporal lobe if bilateral IED was present. Bilateral temporal IED was defined as a 74% or less preponderance in one temporal lobe. Temporal and extratemporal IEDs were indicated when IEDs from ipsi- or contralateral extratemporal regions were present with more than a 25% preponderance to the temporal IED.

#### Ictal EEG classification during scalp EEG recording[13]

A temporal ictal onset zone was defined when the location of the ictal discharges was uni- or bi-temporal, and the amplitude ratio of the temporal vs. parasagittal chain was higher than 2:1 in bipolar montages and higher than 2:1 for the 2 sides in referential montages. A hemispheric ictal onset zone was defined when ictal discharges arose from a lateralized hemisphere and the amplitude ratio of the temporal vs. parasagittal chain was lower than 2:1 in bipolar montages and lower than 2:1 for the two sides in referential montages.

### Neuroimaging studies

#### Brain MRI

MRI was performed using a GE Signa 1.5-Tesla scanner (GE Medical Systems, Inc., Milwaukee, WI, USA) or a 3.0-Tesla scanner (Philips, Best, the Netherlands). All patients underwent the spoiled gradient echo, T2-weighted, and fluid attenuated inversion recovery imaging protocols.[20] The MRI results were classified as 5 subtypes: (a) unilateral HS, (b) bilateral HS, (c) unilateral HS and combined extra-hippocampal lesion (mainly ipsilateral anterior temporal atrophy or diffuse hemiatrophy/focal cerebromalacia), (d) normal, and (e) tumorous lesion involving mesial temporal structures.

#### FDG-PET studies

PET images were obtained (GE Advance PET scanner, GE Medical Systems, Inc.) after patients had fasted for four or more hours and then received an intravenous injection of 7–10 mCi (259–370 MBq) of FDG. EEG during the uptake period demonstrated no EEG seizure activity in any patient. Hypometabolism was determined semi-quantitatively by visual assessment using calibrated color scales with a high or absolute degree of inter-observer agreement. A graduated color scale in 2% increments was used for display and analyses. When the metabolism of the lobe showed a 20% or more reduction compared with the other areas of metabolism, it was regarded as abnormal hypometabolism.[13,21]

### Surgery and outcomes

Intraoperative electrocorticography (ECoG) with subdural electrodes to verify epileptiform discharges was applied in all but 30 patients. If ECoG revealed active spikes in the brain regions adjacent to the standard resection margin, those active regions were supplementally resected.[20,22] We used relatively uniform surgical procedures. Three subtypes of resective operations were performed in our epilepsy center.[20] The first subtype was a standard anterior temporal lobectomy (ATL) with tailored amygdalohippocampectomy (AH) that included the anterior temporal neocortex, as well as a minimal part of the amygdaloid structures and partial hippocampal structures. The second subtype was ATL with a complete resection of the amygdala-hippocampus based on the intraoperative ECoG results. In the third subtype, an additional corticectomy guided by ECoG was added to the second subtype of surgery. The International League Against Epilepsy (ILAE) has recently proposed a simple classification of HS based on semiquantitative cell loss patterns that can be easily used by most laboratories. It identifies three HS types characterized characterized by cell loss affecting all of the sectors of the CA (type1), predominantly CA1 (type 2), or predominantly CA4 (type 3).[23] The resected mesial temporal specimens were analyzed just after surgery, and we classified those histopathologic results according to ILAE classification.

Postoperative seizure outcomes were determined by outpatient clinic interview or telephone interview using Engel’s classification.[24] The patients were instructed to visit the clinic one month after surgery and then every 3 months. If patients became seizure-free, they visited the clinic every six months. For this study, we evaluated the surgical outcomes 2 and 5 years after surgery and at the end of the study period. We also performed year-by-year analyses of surgical outcomes.

### Statistical analyses

To compare seizure-free (Engel I) patients and patients with recurrent seizures (Engel II–IV), we applied Chi-square or Fisher’s exact test for categorical variables. A student’s t-test or the Mann-Whitney U test was performed for continuous variables. Logistic regression analyses were used to verify independent risk factors for seizure recurrence. Variables with p values ≤ 0.05 in the simple logistic regression were tested in the multiple logistic regression analyses. Adjustment for multiple comparisons was done by Fisher’s exact test using the permutation method. Statistical significance was accepted at p < 0.05. The time to first seizure recurrence was plotted using a Kaplan-Meier survival curve to estimate the proportion of individuals with several prognostic factors who remained seizure-free at various time points. A log rank test and a comparison of 95% confidence intervals were used to establish differences between good and bad prognostic factors for seizure recurrence after surgery.

## Results

### Patient characteristics

Demographic and clinical assessment data for subjects are summarized in Table 1. The mean age of seizure onset was 16.9 ± 10.3 years (range 0.2–52). The mean age at surgery was 30.3 ± 10.5 years (range 7–64). 233 (58.3%) patients experienced one or more tonic-clonic seizures before surgery. All patients underwent scalp video EEG and brain MRI. Functional imaging studies were performed in 245 patients (ictal SPECT with/without interictal SPECT) and 336 patients (FDG-PET).

**Table 1:** Demographics and clinical characteristics.

	N = 400
Gender	
Male, n (%)	190 (47.5)
Female, n (%)	210 (52.5)
Age at onset of seizures, year	16.9 ± 10.3
Age at surgery, year	30.3 ± 10.5
< 20, n (%)	57 (14.3)
20–30, n (%)	148 (37.0)
30–40, n (%)	116 (29.0)
> 40, n (%)	79 (19.8)
Duration of epilepsy prior to surgery, year	13.4 ± 8.5
Number of AEDs before surgery	2.44 ± 1.01
Follow-up period after surgery, year	7.6 ± 3.7
Side of surgery	
Right, n (%)	180 (45.0)
Left, n (%)	220 (55.0)
Risk factors	
Febrile seizure, n (%)	155 (38.8)
CNS infection, n (%)	48 (12.0)
Secondarily generalized tonic-clonic seizure, n (%)	233 (58.3)
Aura, n (%)	331 (82.8)

Continuous variables are presented as mean ± SD; categorical variables are presented as N (%). AED, antiepileptic Drug; CNS, central nervous system

### Surgery, surgical outcome, and histopathology

All patients underwent ATL with partial or complete AH. 58 (14.5%) patients received partial AH, of which 38/58 (65.5%) were left sided. 172 (43%) patients showed an interictal spike on the ECoG recording during surgery, of whom 129/172 (75%) underwent a tailored corticectomy. There was no significant difference in surgical outcome between the positive ECoG group that received a corticectomy and the positive ECoG group that did not receive a corticectomy.

All cases were confirmed histopathologically after resective surgeries. We examined postoperative tissue from 382 of 400 MTLE surgeries. HS type could not be defined with certainty for 18 samples. The remaining tissue samples were classed as type1 HS for 288 patients (75.4%), type 2 for 6 patients (1.6%), type 3 for 2 patients (0.5%), and type no-HS for 44 patients (11.5%). 42 patients (11.0%) showed tumors involving the hippocampus without or with hippocampal sclerosis. Among 84 patients with unilateral HS and combined extra-hippocampal lesion, 53 patients showed ipsilesional anterior temporal abnormalities with various degrees of focal cortical dysplasia.

The mean follow-up duration after surgery was 7.6 ± 3.7 years (range, 5.0–21.0 years). The short-term (2-year) outcome data showed that 342 patients (85.5%) were in Engel class I: 337 (84.25%) in class Ia (ILAE class 1).[25] The long- term seizure free rate was good. 334 patients (83.5%) were Engel class I at the fifth year after surgery and 302 patients (79.1%) had an Engel class Ia (ILAE class 1) outcome. Of 13 patients with an Engel class IV two years after surgery, 99.7% (12/13) had persisting seizures at the last follow-up.

### Factors predicting seizure outcomes two and five years after surgery

Subsequent multiple logistic regressions using variables significant in the univariate analyses at year two after surgery showed that tailoredhippocampal resection (OR, 5.295; 95% CI, 1.871–14.986), unilateral HS and combined extra-hippocampal lesion (OR, 3.643; 95% CI, 1.258–10.555), BTH (OR, 3.436; 95% CI, 1.118–9.941), bi-temporal IED (OR, 3.060; 95% CI, 1.389–6.738), and a history of GTC seizures (OR, 2.758; 95% CI, 1.267–6.000) were independently associated with poor outcomes. Significant prognostic factors for poor outcomes at year five after surgery were nearly the same as those at year two, except for bilateral HS on brain MRI (Table 2).

**Table 2:** Simple and multiple logistic regressions for predicting seizure outcome at five year after surgery.

	Univariate OR	P-value	Multivariate OR	P-value
	OR (95% CI)		OR (95% CI)	
duration of epilepsy, year	1.039 (1.008–1.070)	0.014	1.012 (0.968–1.058)	0.594
History of GTC seizure, n (%)	2.645 (1.449–4.827)	0.001	2.318 (1.100–4.886)	0.027
History of Aura, n (%)	0.446 (0.242–0.822)	0.008	0.575 (0.267–1.239)	0.158
MRI findings				
Unilateral HS or HA (reference)				
vs Bilateral HS or HA	5.471 (1.887–15.859)	<0.001	5.471 (1.165–25.043)	0.016
vs Combined extra-hippocampal	4.459 (1.995–9.969	<0.001	5.029 (1.699–14.886)	<0.001
vs Tumorous lesion	1.689 (0.600–4.753)	0.227	1.125 (0.105–12.113)	1.000
vs Normal	1.530 (0.229–10.249)	0.593	1.738 (0.353–8.544)	1.000
PET hypo-metabolism pattern				
Unilateral temporal (mesiobasal) (reference)				
vs Unilateral temporal (extending to posterolateral)	1.875 (0.699–5.027)	0.384	1.490 (0.480–4.626)	1.000
vs Bilateral temporal	5.250 (2.218–12.425)	<0.001	4.438 (1.554–12.678)	0.003
vs Temporal + Extratemporal	4.725 (1.524–14.648)	0.003	3.254 (0.812–13.033)	0.126
Bi-temporal interictal epileptiform	3.559 (1.923–6.588)	<0.001	3.107 (1.422–6.791)	0.004
Partial hippocampal resection	2.054 (1.061–3.979)	0.030	3.450 (1.242–9.587)	0.018

CNS, central nervous system; GTC, generalized tonic-clonic; HS, hippocampal sclerosis; HA, hippocampal atrophy

### Survival analysis of seizure recurrence

Multivariate analyses using the Cox proportional hazards test demonstrated that a history of GTC seizures and tailored hippocampal resection had significantly high hazard ratios (HRs) (HR = 1.711; 95% CI = 1.026–2.855, HR = 2.834; 95% CI = 1.424–5.639, respectively). In the presurgical evaluation, bi-temporal IED (HR = 2.186; 95% CI = 1.288–3.709), BTH (HR = 2.04; 95% CI = 1.138–3.667), bilateral HS (HR = 2.541; 95% CI = 1.217–5.307) and unilateral HS and combined extra-hippocampal lesion (HR = 2.75; 95% CI = 1.589–4.758) were independently associated with seizure recurrence (Table 3).

**Table 3:** Predictors of seizure recurrence (by cox proportional hazards model) of patients with MTLE.

	Univariate anlaysis				Multivariate anlaysis			
	Hazard Ratio	95% CI		P-value	Hazard Ratio	95% CI		P-value
History of GTC seizure, n (%)	2.078	1.275	3.387	0.0034	1.711	1.026	2.855	0.0396
History of Aura, n (%)	0.579	0.353	0.952	0.0311	0.706	0.425	1.173	0.1796
Bi-temporal interictal epileptiform	2.503	1.564	4.008	<0.001	2.186	1.288	3.709	0.0037
MRI findings								
Unilateral HS or HA (reference)		reference				reference		
vs Bilateral HS or HA	2.185	1.099	4.344	0.0258	2.541	1.217	5.307	0.0130
vs Combined extra-hippocampal lesion	2.318	1.394	3.853	0.0012	2.75	1.589	4.758	<0.001
vs Tumorous lesion	1.355	0.415	4.425	0.6149	1.004	0.301	3.35	0.9943
vs Normal	0.983	0.453	2.133	0.9650	0.927	0.371	2.321	0.8719
PET hypo-metabolism pattern								
Unilateral temporal (mesiobasal) (reference)		reference				reference		
vs Unilateral temporal (extending to posterolateral)	1.696	0.921	3.125	0.0900	1.358	0.725	2.541	0.3390
vs Bilateral temporal	2.791	1.641	4.746	<0.001	2.043	1.138	3.667	0.0167
vs Temporal + Extratemporal	2.34	1.093	5.01	0.0286	1.472	0.661	3.277	0.3436
Partial hippocampal resection	1.45	0.828	2.54	0.1934	2.834	1.424	5.639	0.0030

CNS, central nervous system; GTC, generalized tonic-clonic; HS, hippocampal sclerosis; HA, hippocampal atrophy

**Fig 1:**
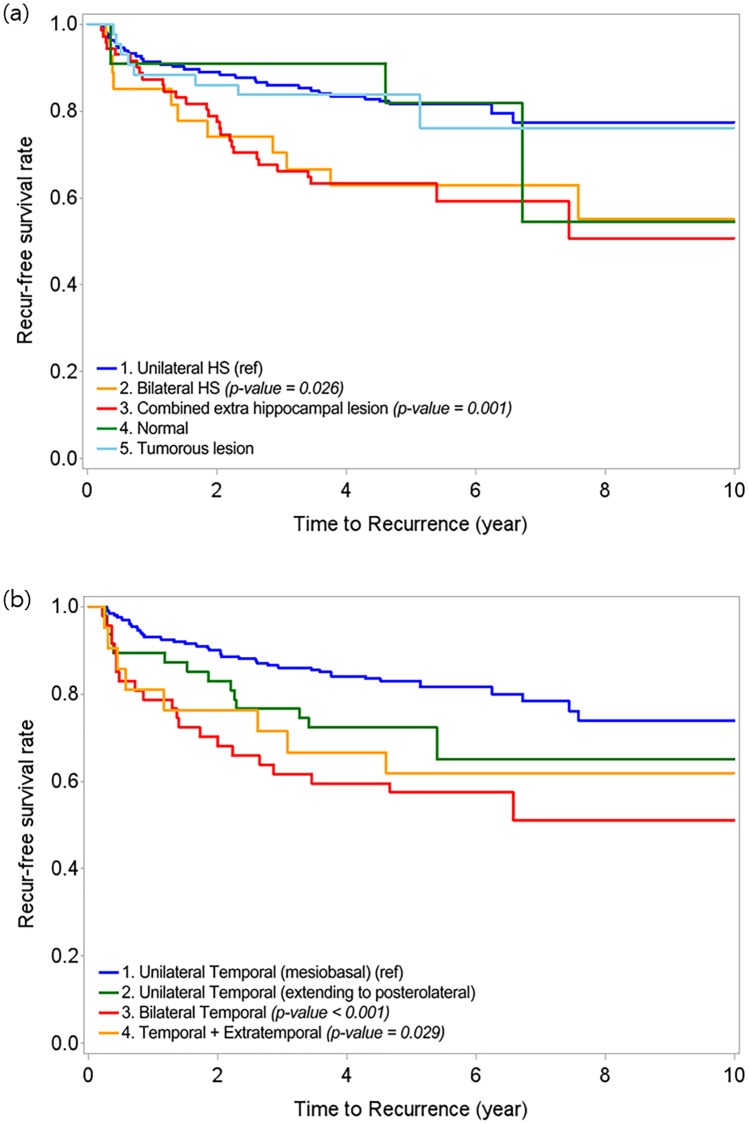
Kaplan-Meier survival curves according to (a) MRI findings and (b) PET hypometabolism patterns. (a) The Kaplan-Meier survival curves show statistically significant associations of bilateral HS and unilateral HS combined with extra-hippocampal lesion with seizure recurrence. (b) The survival curves show significantly more frequent recurrence in patients with BTH or temporal plus extratemporal hypometabolism compared with patients with UTH involving only mesial temporal and temporal pole structures. HS, hippocampal sclerosis; UTH, unilateral temporal hypometabolism; BTH, bilateral temporal hypometabolism.

We used a Kaplan Meier’s survival analysis to compare seizure-free survival according to different MRI findings and PET hypometabolism patterns. The survival curves show a significantly higher seizure-free ratio in patients with unilateral HS compared with bilateral HS or unilateral HS combined with extra-hippocampal lesion (log-rank test, p = 0.026 and p = 0.001, respectively) (Fig 1). In addition, patients with BTH (log-rank test, p < 0.001) and temporal plus extratemporal hypometabolism (log-rank test, p = 0.029) showed seizure recurrence more frequently than patients with UTH involving only mesial temporal and temporal pole structures (Fig 1).

### Subgroup analysis

The subgroup analysis was performed on 208 patients with only unilateral HS. Five years after surgery, BTH (OR, 5.838; 95% CI, 1.195–28.532) and tailoredhippocampal resection (OR, 11.053; 95% CI, 2.306–52.971) independently predicted poor outcomes in the multiple logistic regression (Table 4).

**Table 4:** Subgroup analysis of seizure outcome at five year after surgery in patients with unilateral HS.

	Univariate OR	P-value	Multivariate OR	P-value
	OR (95% CI)		OR (95% CI)	
Preoperative duration of epilepsy, year	1.064 (1.006–1.124)	0.029	1.040 (0.970–1.114)	0.270
PET hypo-metabolism pattern				
Unilateral temporal (mesiobasal) (reference)				
vs Unilateral temporal (extending to posterolateral)	1.321 (0.184–9.480)	>0.999	2.163 (0.260–17.985)	>0.999
vs Bilateral temporal	4.162 (1.013–17.107)	0.048	5.838 (1.195–28.532)	0.024
vs Temporal + Extratemporal	4.625 (0.547–39.139)	>0.999	3.626 (0.336–39.126)	0.588
Partial hippocampal resection	8.080 (2.332–27.996)	< 0.001	11.053 (2.306–52.971)	0.004

CNS, central nervous system; GTC, generalized tonic-clonic; HS, hippocampal sclerosis; HA, hippocampal atrophy

## Discussion

We observed surgical outcomes and verified possible prognostic factors in a large group of patients with MTLE during a long-term follow-up period. We found that 85.5% of patients experienced seizure freedom 2 years after MTLE surgery. In the further follow-up period (5 years after surgery) the rate of seizure freedom declined slightly to 83.5%. We demonstrated that neuro-radiological findings, including unilateral HS combined with extra-hippocampal abnormalities, bilateral HS on MRI, and BTH on FDG-PET were independently associated with seizure recurrence. Furthermore, in the subgroup with unilateral HS, BTH predicted a poor long-term outcome.

In past studies, the presence of secondarily generalized seizures before surgery was closely related to postsurgical seizure recurrence,[2,4,5] and some previous studies showed that a long duration of epilepsy was associated with worse outcomes after surgery.[2,26] However, other studies did not find those associations, which has yielded controversy in the literature.[6,7] Among the clinical variables we considered in this study, only the presence of preoperative secondarily generalized seizures had a significant association with short- and long-term outcomes. The duration of epilepsy did not affect outcomes. Our results might stem from the relatively shorter epilepsy duration before surgery among our study population compared with previous studies.

Advances in imaging technologies have improved the detection of brain structural and functional changes related to TLE.[27–29] Extrahippocampal abnormalities in MTLE are not uncommon.[30–34] TLE is a common progressive disease in which the persistent paroxysmal activity during seizures alters highly diffuse, often bilateral, structural brain connectivity. The degree of hippocampal and extrahippocampal brain atrophy might be related to the severity and duration of epilepsy.[31,35] Poor surgical outcomes have been associated with a widespread pattern of preoperative gray matter atrophy in MTLE patients.[36] In our study, unilateral HS combined with extra-hippocampal lesions and bilateral HS on MRI were independently associated with seizure recurrence, compared with unilateral HS. 53 patients (65.5%) with extrahippocampal lesions showed ipsilateral anterior temporal lesions with focal cortical dysplasia in their pathology reports. This finding suggests that the involvement of extrahippocampal structures could be evidence of widespread seizure activity propagation or other aspects of the epileptogenic process.[34,37,38]

Previous studies demonstrated that non-lesional FDG-PET positive patients had outcomes comparable to those of lesional cases after epilepsy surgery.[10,39,40] In our study, 15 patients with MRI-negative TLE who underwent resective surgery experienced favorable results. 86.7% of patients (13 out of 15) with UTH on the preoperative FDG-PET study were seizure free at the 5^th^ year after surgery. But the remaining 2 patients, who had temporal plus extratemporal hypometabolism and BTH on their PET studies, had poor outcomes.

The extent of interictal hypometabolism on FDG-PET could be related to the neuronal networks involved by ictal discharge generation and spread pathways.[41] Although our study did not reveal differences in surgical outcomes between patients with unilateral hypometabolism restricted to the mesiobasal temporal regions and those in whom it extended to the posterolateral temporal region, we did find that BTH was independently associated with seizure recurrence. Some studies looked at functional connectivity in epileptogenic temporal lobe networks and reported that patients with left or right MTLE presented important decreases in connectivity with the contralateral mesial temporal structures.[42–44] In our subgroup analysis of patients with unilateral HS, the patients with BTH presented poor surgical outcomes. Hippocampal change on brain MRI does not appear to determine the existence of metabolic reduction, which suggests that metabolic changes on FDG-PET could precede structural alterations by indicating decreased functional connectivity, and that could be related to more profound neuronal dysfunction.

As one of the complications following anterior temporal lobe surgery, many patients experience various degrees of verbal or non-verbal memory decline.[45] Still, some previous studies showed that the resection extent of the mesial temporal structure correlated positively with surgical outcomes,[46–48] which is in agreement with our results. We found that tailored hippocampal resection was independently associated with seizure recurrence after surgery, even though it was based on intraoperative ECoG results. In contrast, one study found that patients who underwent tailored AH had outstanding outcomes compared with standard surgical procedures, with 66.9% of patients free from disabling seizures (Engel I) and low surgery-related complications.[26]

The distribution of HS types was different from that reported in some studies[49,50], and confirmed that type 1 is the most frequent, whereas CA4-predominant type 3 was rare. Similar to previous study[49], we found no correlation between postoperative outcome and the ILAE hippocampal sclerosis pattern. One recent study has reported the patients with type 2 HS had generally better long-term outcomes than those with type 1.[50] However, we did not observe the similar result that may be due to very small sample sized of type 2 HS in our cases.

We hypothesized that the long and short-term outcomes might be influenced by different factors: seizure recurrence many years after surgery might be associated with different risk factors than those associated with early seizure recurrence.[4] It was interesting that the prognostic factors for short- and long-term outcomes had no difference without bilateral HS on MRI in the present study. In our results, the overall seizure-free rate is excellent, with little difference between the 2^nd^ and 5^th^ year outcomes (Engel class I, 85.5% at the 2^nd^ year after surgery and 83.5% at the 5^th^ year after surgery). Most Engel class IV patients at the 2^nd^ year (12/13, 99.7%) had persisting seizures in the last year of follow-up, which might explain the similarity of our short- and long-term prognostic factors. Although bilateral metabolic reduction was independently associated with poor outcomes in both the short- and long-terms, bilateral HS could not predict short-term poor outcomes. Therefore, BTH might be a more valuable factor for predicting surgical outcome than bilateral HS.

This study has limitations. Our study design was retrospective, with the potential for incomplete data and the bias of unmeasured confounders. We tried to minimize the missing data by applying the same standardized set of presurgical evaluations to patients. The PET images were analyzed semi-quantitatively in a visual analysis, but the degree of inter-observer agreement was high or absolute.

## Conclusions

We found that a combination of multiple tests provided highly precise localization of seizure focus and eventually better surgical outcomes. Our results further support the notion that in patients with MTLE and accompanying BTH on FDG-PET but without bilateral MRI changes, surgical suitability should be proved by a more comprehensive work-up. Also, our results might suggest that PET imaging has clinical effectiveness even with concordance between the MRI and electrophysiological data.

## Supporting information

S1 Table Demographics of subjects. Demographic and clinical assessment data for subjects are included.
(XLSX)

## References

[1] Uijl SG, Leijten FS, Arends JB, Parra J, van Huffelen AC, Moons KG. Prognosis after temporal lobe epilepsy surgery: the value of combining predictors. Epilepsia. 2008; 49:1317–23. 10.1111/j.1528-1167.2008.01695.x 18557776

[2] Janszky J, Janszky I, Schulz R, Hoppe M, Behne F, Pannek HW, et al Temporal lobe epilepsy with hippocampal sclerosis: predictors for long-term surgical outcome. Brain. 2005; 128:395–404. 10.1093/brain/awh358 15634733

[3] McIntosh AM, Wilson SJ, Berkovic SF. Seizure outcome after temporal lobectomy: current research practice and findings. Epilepsia. 2001; 42:1288–307. 11737164 10.1046/j.1528-1157.2001.02001.x

[4] McIntosh AM, Kalnins RM, Mitchell LA, Fabinyi GC, Briellmann RS, Berkovic SF. Temporal lobectomy: long-term seizure outcome, late recurrence and risks for seizure recurrence. Brain. 2004; 127:2018–30. 10.1093/brain/awh221 15215219

[5] Schwartz TH, Jeha L, Tanner A, Bingaman W, Sperling MR. Late seizures in patients initially seizure free after epilepsy surgery. Epilepsia. 2006; 47:567–73. 10.1111/j.1528-1167.2006.00469.x 16529623

[6] Elsharkawy AE, Alabbasi AH, Pannek H, Oppel F, Schulz R, Hoppe M, et al Long-term outcome after temporal lobe epilepsy surgery in 434 consecutive adult patients. J Neurosurg. 2009; 110:1135–46. 10.3171/2008.6.jns17613 19025359

[7] Lowe NM, Eldridge P, Varma T, Wieshmann UC. The duration of temporal lobe epilepsy and seizure outcome after epilepsy surgery. Seizure. 2010; 19:261–3. 10.1016/j.seizure.2010.02.011 20430656

[8] Keller SS, Cresswell P, Denby C, Wieshmann U, Eldridge P, Baker G, et al Persistent seizures following left temporal lobe surgery are associated with posterior and bilateral structural and functional brain abnormalities. Epilepsy Res. 2007; 74:131–9. 10.1016/j.eplepsyres.2007.02.005 17412561

[9] Schijns OE, Bien CG, Majores M, von Lehe M, Urbach H, Becker A, et al Presence of temporal gray-white matter abnormalities does not influence epilepsy surgery outcome in temporal lobe epilepsy with hippocampal sclerosis. Neurosurgery. 2011; 68:98–106; discussion 7. 10.1227/NEU.0b013e3181fc60ff 21150756

[10] LoPinto-Khoury C, Sperling MR, Skidmore C, Nei M, Evans J, Sharan A, et al Surgical outcome in PET-positive, MRI-negative patients with temporal lobe epilepsy. Epilepsia. 2012; 53:342–8. 10.1111/j.1528-1167.2011.03359.x 22192050

[11] Choi JY, Kim SJ, Hong SB, Seo DW, Hong SC, Kim BT, et al Extratemporal hypometabolism on FDG PET in temporal lobe epilepsy as a predictor of seizure outcome after temporal lobectomy. Eur J Nucl Med Mol Imaging. 2003; 30:581–7. 10.1007/s00259-002-1079-8 12557048

[12] Blum DE, Ehsan T, Dungan D, Karis JP, Fisher RS. Bilateral temporal hypometabolism in epilepsy. Epilepsia. 1998; 39:651–9. 9637608 10.1111/j.1528-1157.1998.tb01434.x

[13] Joo EY, Lee EK, Tae WS, Hong SB. Unitemporal vs bitemporal hypometabolism in mesial temporal lobe epilepsy. Arch Neurol. 2004; 61:1074–8. 10.1001/archneur.61.7.1074 15262738

[14] Ho SS, Newton MR, McIntosh AM, Kalnins RM, Fabinyi GC, Brazenor GA, et al Perfusion patterns during temporal lobe seizures: relationship to surgical outcome. Brain. 1997; 120 (Pt 11):1921–8. 9397011 10.1093/brain/120.11.1921

[15] Tae WS, Joo EY, Kim JH, Han SJ, Suh YL, Kim BT, et al Cerebral perfusion changes in mesial temporal lobe epilepsy: SPM analysis of ictal and interictal SPECT. Neuroimage. 2005; 24:101–10. 10.1016/j.neuroimage.2004.08.005 15588601

[16] Na M, Ge H, Shi C, Shen H, Wang Y, Pu S, et al Long-term seizure outcome for international consensus classification of hippocampal sclerosis: a survival analysis. Seizure. 2015; 25:141–6. 10.1016/j.seizure.2014.10.006 25455728

[17] Jeong SW, Lee SK, Hong KS, Kim KK, Chung CK, Kim H. Prognostic factors for the surgery for mesial temporal lobe epilepsy: longitudinal analysis. Epilepsia. 2005; 46:1273–9. 10.1111/j.1528-1167.2005.33504.x 16060939

[18] Joo EY, Hong SB, Lee EK, Tae WS, Kim JH, Seo DW, et al Regional cerebral hyperperfusion with ictal dystonic posturing: ictal-interictal SPECT subtraction. Epilepsia. 2004; 45:686–9. 10.1111/j.0013-9580.2004.35003.x 15144436

[19] Cho JW, Hong SB, Lee JH, Kang JW, Lee MJ, Lee JY, et al Contralateral hyperperfusion and ipsilateral hypoperfusion by ictal SPECT in patients with mesial temporal lobe epilepsy. Epilepsy Res. 2010; 88:247–54. 10.1016/j.eplepsyres.2009.12.002 20092979

[20] Joo EY, Han HJ, Lee EK, Choi S, Jin JH, Kim JH, et al Resection extent versus postoperative outcomes of seizure and memory in mesial temporal lobe epilepsy. Seizure. 2005; 14:541–51. 10.1016/j.seizure.2005.08.011 16242970

[21] Joo EY, Hong SB, Han HJ, Tae WS, Kim JH, Han SJ, et al Postoperative alteration of cerebral glucose metabolism in mesial temporal lobe epilepsy. Brain. 2005; 128:1802–10. 10.1093/brain/awh534 15872014

[22] Wiebe S, Blume WT, Girvin JP, Eliasziw M. A randomized, controlled trial of surgery for temporal-lobe epilepsy. N Engl J Med. 2001; 345:311–8. 10.1056/nejm200108023450501 11484687

[23] Blumcke I, Thom M, Aronica E, Armstrong DD, Bartolomei F, Bernasconi A, et al International consensus classification of hippocampal sclerosis in temporal lobe epilepsy: a Task Force report from the ILAE Commission on Diagnostic Methods. Epilepsia. 2013; 54:1315–29. 10.1111/epi.12220 23692496

[24] Engel J Jr, V NP, Rasmussen TB, Ojemann LM. Outcome with respect to epileptic seizures In Engel J Jr, ed. Surgical treat-ment of the epilepsies New York: Raven Press 1993:609–21.

[25] Wieser HG, Blume WT, Fish D, Goldensohn E, Hufnagel A, King D, et al ILAE Commission Report. Proposal for a new classification of outcome with respect to epileptic seizures following epilepsy surgery. Epilepsia. 2001; 42:282–6. 11240604

[26] Wieser HG, Ortega M, Friedman A, Yonekawa Y. Long-term seizure outcomes following amygdalohippocampectomy. J Neurosurg. 2003; 98:751–63. 10.3171/jns.2003.98.4.0751 12691400

[27] Liu M, Chen Z, Beaulieu C, Gross DW. Disrupted anatomic white matter network in left mesial temporal lobe epilepsy. Epilepsia. 2014; 55:674–82. 10.1111/epi.12581 24650167

[28] Bernhardt BC, Bonilha L, Gross DW. Network analysis for a network disorder: The emerging role of graph theory in the study of epilepsy. Epilepsy Behav. 2015; 50:162–70. 10.1016/j.yebeh.2015.06.005 26159729

[29] Engel J Jr., Thompson PM, Stern JM, Staba RJ, Bragin A, Mody I. Connectomics and epilepsy. Curr Opin Neurol. 2013; 26:186–94. 10.1097/WCO.0b013e32835ee5b8 23406911 PMC4064674

[30] Labate A, Cerasa A, Aguglia U, Mumoli L, Quattrone A, Gambardella A. Voxel-based morphometry of sporadic epileptic patients with mesiotemporal sclerosis. Epilepsia. 2010; 51:506–10. 10.1111/j.1528-1167.2009.02310.x 19780790

[31] Labate A, Cherubini A, Tripepi G, Mumoli L, Ferlazzo E, Aguglia U, et al White matter abnormalities differentiate severe from benign temporal lobe epilepsy. Epilepsia. 2015; 56:1109–16. 10.1111/epi.13027 26096728

[32] Bonilha L, Elm JJ, Edwards JC, Morgan PS, Hicks C, Lozar C, et al How common is brain atrophy in patients with medial temporal lobe epilepsy? Epilepsia. 2010; 51:1774–9. 10.1111/j.1528-1167.2010.02576.x 20412283

[33] Bonilha L, Rorden C, Appenzeller S, Coan AC, Cendes F, Li LM. Gray matter atrophy associated with duration of temporal lobe epilepsy. Neuroimage. 2006; 32:1070–9. 10.1016/j.neuroimage.2006.05.038 16872843

[34] Alvim MK, Coan AC, Campos BM, Yasuda CL, Oliveira MC, Morita ME, et al Progression of gray matter atrophy in seizure-free patients with temporal lobe epilepsy. Epilepsia. 2016; 57:621–9. 10.1111/epi.13334 26865066

[35] Coan AC, Appenzeller S, Bonilha L, Li LM, Cendes F. Seizure frequency and lateralization affect progression of atrophy in temporal lobe epilepsy. Neurology. 2009; 73:834–42. 10.1212/WNL.0b013e3181b783dd 19752449

[36] Yasuda CL, Valise C, Saude AV, Pereira AR, Pereira FR, Ferreira Costa AL, et al Dynamic changes in white and gray matter volume are associated with outcome of surgical treatment in temporal lobe epilepsy. Neuroimage. 2010; 49:71–9. 10.1016/j.neuroimage.2009.08.014 19683060

[37] Levesque MF, Nakasato N, Vinters HV, Babb TL. Surgical treatment of limbic epilepsy associated with extrahippocampal lesions: the problem of dual pathology. J Neurosurg. 1991; 75:364–70. 10.3171/jns.1991.75.3.0364 1869934

[38] Li LM, Cendes F, Andermann F, Watson C, Fish DR, Cook MJ, et al Surgical outcome in patients with epilepsy and dual pathology. Brain. 1999; 122 (Pt 5):799–805. 10355666 10.1093/brain/122.5.799

[39] Gok B, Jallo G, Hayeri R, Wahl R, Aygun N. The evaluation of FDG-PET imaging for epileptogenic focus localization in patients with MRI positive and MRI negative temporal lobe epilepsy. Neuroradiology. 2013; 55:541–50. 10.1007/s00234-012-1121-x 23223825

[40] Capraz IY, Kurt G, Akdemir O, Hirfanoglu T, Oner Y, Sengezer T, et al Surgical outcome in patients with MRI-negative, PET-positive temporal lobe epilepsy. Seizure. 2015; 29:63–8. 10.1016/j.seizure.2015.03.015 26076845

[41] Chassoux F, Semah F, Bouilleret V, Landre E, Devaux B, Turak B, et al Metabolic changes and electro-clinical patterns in mesio-temporal lobe epilepsy: a correlative study. Brain. 2004; 127:164–74. 10.1093/brain/awh014 14534161

[42] Pittau F, Grova C, Moeller F, Dubeau F, Gotman J. Patterns of altered functional connectivity in mesial temporal lobe epilepsy. Epilepsia. 2012; 53:1013–23. 10.1111/j.1528-1167.2012.03464.x 22578020 PMC3767602

[43] Pereira FR, Alessio A, Sercheli MS, Pedro T, Bilevicius E, Rondina JM, et al Asymmetrical hippocampal connectivity in mesial temporal lobe epilepsy: evidence from resting state fMRI. BMC Neurosci. 2010; 11:66 10.1186/1471-2202-11-66 20525202 PMC2890013

[44] Morgan VL, Abou-Khalil B, Rogers BP. Evolution of functional connectivity of brain networks and their dynamic interaction in temporal lobe epilepsy. Brain Connect. 2015; 5:35–44. 10.1089/brain.2014.0251 24901036 PMC4313394

[45] Lambon Ralph MA, Ehsan S, Baker GA, Rogers TT. Semantic memory is impaired in patients with unilateral anterior temporal lobe resection for temporal lobe epilepsy. Brain. 2012; 135:242–58. 10.1093/brain/awr325 22287382 PMC3267985

[46] Awad IA, Katz A, Hahn JF, Kong AK, Ahl J, Luders H. Extent of resection in temporal lobectomy for epilepsy. I. Interobserver analysis and correlation with seizure outcome. Epilepsia. 1989; 30:756–62. 2591342 10.1111/j.1528-1157.1989.tb05335.x

[47] Nayel MH, Awad IA, Luders H. Extent of mesiobasal resection determines outcome after temporal lobectomy for intractable complex partial seizures. Neurosurgery. 1991; 29:55–60; discussion -1. 1870688 10.1097/00006123-199107000-00009

[48] Wyler AR, Hermann BP, Somes G. Extent of medial temporal resection on outcome from anterior temporal lobectomy: a randomized prospective study. Neurosurgery. 1995; 37:982–90; discussion 90–1. 8559349 10.1227/00006123-199511000-00019

[49] Deleo F, Garbelli R, Milesi G, Gozzo F, Bramerio M, Villani F, et al Short- and long-term surgical outcomes of temporal lobe epilepsy associated with hippocampal sclerosis: Relationships with neuropathology. Epilepsia. 2016; 57:306–15. 10.1111/epi.13277 26676889

[50] Mathon B, Bielle F, Samson S, Plaisant O, Dupont S, Bertrand A, et al Predictive factors of long-term outcomes of surgery for mesial temporal lobe epilepsy associated with hippocampal sclerosis. Epilepsia. 2017; 58:1473–85. 10.1111/epi.13831 28656696

